# Investigation of the Microstructure Evolution in a Fe-17Mn-1.5Al-0.3C Steel via In Situ Synchrotron X-ray Diffraction during a Tensile Test

**DOI:** 10.3390/ma10101129

**Published:** 2017-09-25

**Authors:** Yan Ma, Wenwen Song, Wolfgang Bleck

**Affiliations:** Steel Institute, RWTH Aachen University, Intzestraße 1, 52072 Aachen, Germany; wenwen.song@iehk.rwth-aachen.de (W.S.); bleck@iehk.rwth-aachen.de (W.B.)

**Keywords:** high-Mn steel, TRIP effect, TWIP effect, strain-hardening behavior, synchrotron X-ray diffraction, stacking fault energy, mechanical properties, microstructure

## Abstract

The quantitative characterization of the microstructure evolution in high-Mn steel during deformation is of great importance to understanding its strain-hardening behavior. In the current study, in situ high-energy synchrotron X-ray diffraction was employed to characterize the microstructure evolution in a Fe-17Mn-1.5Al-0.3C steel during a tensile test. The microstructure at different engineering strain levels—in terms of ε-martensite and α’-martensite volume fractions, the stacking fault probability, and the twin fault probability—was analyzed by the Rietveld refinement method. The Fe-17Mn-1.5Al-0.3C steel exhibits a high ultimate tensile strength with a superior uniform elongation and a high strain-hardening rate. The remaining high strain-hardening rate at the strain level about 0.025 to 0.35 results from ε-martensite dominant transformation-induced-plasticity (TRIP) effect. The increase in the strain-hardening rate at the strain level around 0.35 to 0.43 is attributed to the synergetic α’-martensite dominant TRIP and twinning-induced-plasticity (TWIP) effects. An evaluation of the stacking fault energy (SFE) of the Fe-17Mn-1.5Al-0.3C steel by the synchrotron measurements shows good agreement with the thermodynamic calculation of the SFE.

## 1. Introduction

In the last three decades, the requirement for advanced high-strength steels (AHSS) has been highlighted in the automobile industry to reduce automobile weight and fuel consumption, as well as to improve passenger safety [1]. To obtain an excellent combination of high strength and superior ductility has become the top issue in the modern AHSS design for automotive applications. With the aid of the strengthening mechanisms like dislocation strengthening, solid solution strengthening, and precipitation hardening, the strength can be increased, albeit at the expense of ductility. Taking advantage of additional strengthening mechanisms, such as the transformation-induced-plasticity (TRIP) effect [2,3,4,5], twinning-induced-plasticity (TWIP) effect [2,3,4,5,6], and microband-induced-plasticity (MBIP) effect [4,7,8], high-Mn steels have been developed to overcome the strength-ductility paradox in conventional high strength steels. Consequently, the high-Mn steels with austenitic microstructure manifest both high strength and outstanding ductility.

Stacking fault energy (SFE) plays a decisive role in controlling the mechanical properties and strain-hardening behavior in the high-Mn austenitic steels. When the SFE is below ~20 mJ/m^2^, the high-Mn steels are deformed by dislocation slip and ε-/α’-martensitic transformation, and the TRIP effect is the dominant deformation mechanism [9,10,11]. High-Mn steels with a SFE in the range of 20–50 mJ/m^2^ exhibit TWIP effect in combination with dislocation slip during plastic deformation [4,11]. The planar glide of dislocations occurs when the SFE of high-Mn steels is greater than 50 mJ/m^2^ [4,11]. The TWIP effect in high-Mn steels has been intensively studied since the 2000s because of its effective contribution to the improvement of the strain-hardening rate and the retardation of necking. It is believed that nanoscale deformation twins contribute to the progressive refinement of the microstructure and the twin boundaries act as obstacles to the dislocation movement [6,12,13]. The strain-hardening in TWIP steels is attributed to the so-called ‘dynamic Hall-Petch effect’ [14,15,16]. On the contrary, due to the challenge to obtain an appropriate martensite transformation rate during plastic deformation, the TRIP effect is generally expected to be a less effective strengthening mechanism in high-Mn steels [17].

In some high-Mn steels with a SFE value about 20 mJ/m^2^, the TRIP and TWIP effects occur simultaneously or in a sequence during plastic deformation, and are able to effectively promote a high strain-hardening capacity [18,19]. The very fine microstructure in TRIP/TWIP steels has been characterized by transmission electron microscopy (TEM) [18,19]. However, due to the limitation of this technique, only very local features at nanometer scale in the material can be detected. The statistical information of the microstructure in a larger volume at millimeter scale is highly needed in order to quantitatively explain the deformation behavior of the material. Due to the unique properties of synchrotron X-ray diffraction (SYXRD), this state-of-the-art technique has been widely used as a powerful characterization tool in various material research over the last two decades [20,21,22], especially for the AHSS with sophisticated microstructure [23,24]. SYXRD has been successfully applied to the investigation on the phase transformation [25,26,27,28], precipitation behavior [8,29], residual stress [30], and texture features [25,31] in a large number of steel grades. The high energy of synchrotron X-ray enables high penetration depth and fast measurement for many materials [22]. The high penetration depth results in a considerably large detection volume, which provides fine statistical information [32]; while the high flux of the synchrotron X-ray and the advanced detection techniques allow the in situ investigation [22,23]. With the aid of this advanced materials characterization method, it is able to in situ monitor the microstructure evolution in high-Mn steels during deformation, which is of great significance to understanding the deformation mechanisms.

In the present study, the microstructure evolution in a Fe-17Mn-1.5Al-0.3C TRIP/TWIP steel during a tensile deformation was characterized by the in situ SYXRD method. The SFE of the material was further evaluated. The quantitative microstructure analysis provides a better understanding of the strain-hardening mechanisms of the TRIP/TWIP steel.

## 2. Material and Methods

### 2.1. Materials and Processing

**Figure 1:**
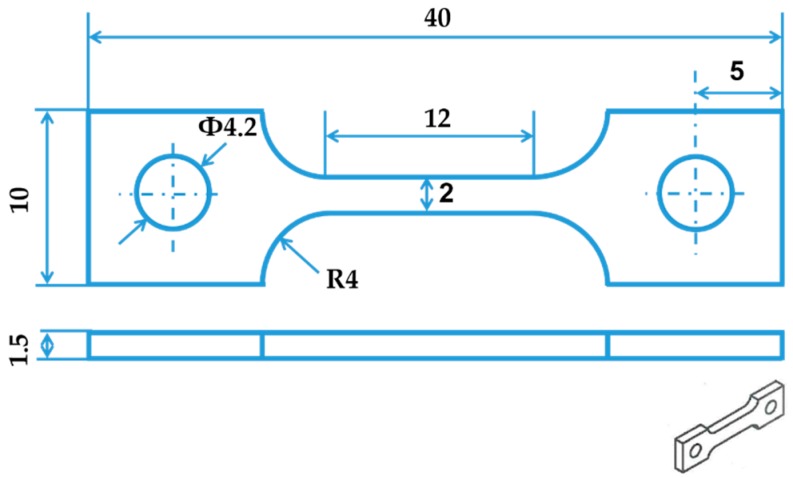
Geometry of the tensile specimen, in mm.

The chemical composition of the investigated Fe-17Mn-1.5Al-0.3C steel is listed in Table 1. The steel was melted in an induction furnace and cast into an ingot with about 80 kg weight. Hot forging was then carried out at 1423 K (1150 °C). The forged ingot was then homogenized at 1423 K (1150 °C) for 5 h, followed by air cooling. The segregation of alloying elements, especially the highly-alloyed Mn, was significantly reduced. Subsequently, the forged ingot experienced the hot rolling and cold rolling to steel sheets with a final thickness of 1.5 mm. The tensile test samples were cut from the cold rolled steel sheets with a geometry shown in Figure 1. Finally, a recrystallization annealing was conducted on the investigated material at 1173 K (900 °C) for 20 min with a heating rate of ~30 K/s, and then the material was quenched in water. Before the tensile test, the sample surface was ground with SiC paper (360 grit) to remove the oxidation layer on the surface. Optical microscopic observation of the samples before and after deformation was performed with an electropolishing method [4]. The Klemm etchant was used for better illustration of the microstructure features, e.g., grain boundaries, twin boundaries, etc. The microstructure was investigated with a magnification of 500×.

### 2.2. In Situ Tensile Test Tracked by Synchrotron X-ray Diffraction

**Figure 2:**
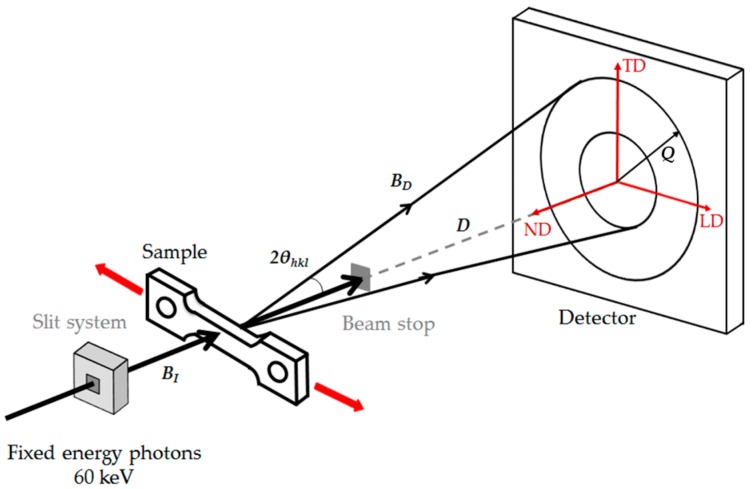
Schematic illustration of the setup of the in situ tensile test tracked by SYXRD. *B_I_* and *B_D_* are the incident and diffracted beams, respectively; *D* is the detector distance between the tensile sample and the fast area detector; 2θ*_hkl_* is the angle between the incident and diffracted beams; *Q* is the scattering vector; LD is the loading direction, TD is the transverse direction and ND is the normal direction.

The high-energy SYXRD experiment was conducted at beamline P02.1 of PETRA III in Deutsches Elektronen-Synchrotron (DESY) center (Bahrenfeld, Hamburg, Germany). The beamline was operated at a fixed energy of 60 keV, supplying a hard X-ray with a wavelength of 0.20707 Å. The high photon energy and flux of the beamline P02.1 enable time-resolved characterization on the structural transformation in a sub-second regime [33]. Figure 2 illustrates the setup of the in situ tensile test tracked by the synchrotron X-ray schematically. The dog-bone shaped tensile test specimen shown in Figure 1 was located between the incident beam and the fast area detector (PerkinElmer XRD1621) (PerkinElmer Optoelectronics, Fremont, CA, USA) [33], which was placed at a distance of about 1 m from the tensile sample. The tensile test was performed at room temperature at a strain rate of 1 × 10^−3^ s^−1^. The incident beam with dimensions of 0.6 mm × 0.6 mm was perpendicular to the tensile specimen and penetrated through the center of the sample gauge section. Two-dimensional diffraction patterns during the tensile test were collected by the fast area detector every 5 s. A measurement of the standard CeO_2_ powder sample was conducted to calibrate the detector distance (*D*) and the instrument broadening. Fit2D software [34] was used to integrate the collected two-dimensional diffraction patterns over 360° into intensity-scattering vector *Q* plots.

### 2.3. Quantitative Microstructure Analysis by the Rietveld Refinement Method

**Figure 3:**
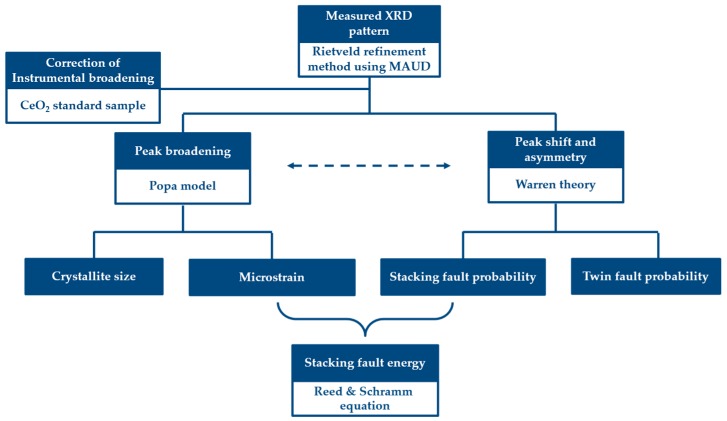
The flow chart of the SYXRD profile-analysis procedures.

The SYXRD whole profiles were analyzed by the Rietveld refinement method with the aid of Materials Analysis Using Diffraction (MAUD) software [35]. For the accurate profile broadening analysis, the pre-analysis on the instrumental broadening was conducted by using the CeO_2_ standard sample under the same experimental conditions as the SYXRD measurements during the tensile test. The instrumental broadening was subsequently subtracted for every profile analysis. The quantitative microstructure study in present work was achieved by the combination of the Popa model [36] and the Warren theory [37,38], which are implemented in the MAUD code as ‘Popa rules’ for the size-strain model and ‘Warren’ for the planar defect model, respectively. The flow chart of the SYXRD profile-analysis procedures is illustrated in Figure 3.

According to the Popa model [36], the profile broadening is associated with microstrain and crystallite size of the material. When the contributions of the microstrain and crystallite size to the X-ray diffraction (XRD) peak profiles are taken into account, the Voigt function in reciprocal space can be expressed as follows:

(1) $$V_{H}\left(Q\right)=\int^{\text{​}}d\left(∆H\right)L_{H}\left(Q+2\pi ∆H\right)G_{H}\left(∆H\right)$$

(2) $$L_{H}\left(Q\right)=\left(3\langle R_{h}\rangle /4\pi \right){\left[1+9{\langle R_{h}\rangle }^{2}{\left(Q-2\pi H\right)}^{2}/16\right]}^{-1}$$

(3) $$G_{H}\left(∆H\right)={\left(2\pi \langle \varepsilon_{hh}^{2}\rangle \right)}^{-1/2}H^{-1}exp\left[-{\left(∆H\right)}^{2}/\left(2H^{2}\langle \varepsilon_{hh}^{2}\rangle \right)\right]$$

where, Q is the wave-vector transfer, H is the reciprocal-lattice vector, $\langle R_{h}\rangle$ represents the average crystallite size in the crystal direction h, and the quantity $\langle \varepsilon_{hh}\rangle$ denotes the (hkl)-dependent strain.

In the present study, the average crystallite size and the (hkl)-dependent strain model for the cubic Laue group are formulated as follows [36]:

(4) $$\langle R_{h}\rangle =R_{0}+R_{1}K_{4}^{1}\left(x,\varphi \right)+R_{2}K_{6}^{1}\left(x,\varphi \right)+\ldots$$

(5) $$\langle \varepsilon_{hh}^{2}\rangle E_{H}^{4}=E_{1}\left(h^{4}+k^{4}+l^{4}\right)+2E_{2}\left(h^{2}k^{2}+k^{2}l^{2}+l^{2}h^{2}\right)$$

where, $K_{2l}^{\mu }\left(x,\varphi \right)$ is the symmetrized harmonics for the cubic group, which are listed below in Equations (6)–(8), and $E_{1}$ and $E_{2}$ are coefficients:

(6) $$K_{4}^{1}\left(x,\varphi \right)=0.3046972P_{4}^{0}\left(x\right)+0.3641828P_{4}^{4}\left(x\right)\cos4\varphi$$

(7) $$K_{6}^{1}\left(x,\varphi \right)=-0.1410474P_{6}^{0}\left(x\right)+0.527751P_{6}^{4}\left(x\right)\cos4\varphi$$

(8) $$K_{6}^{2}\left(x,\varphi \right)=-0.4678013P_{6}^{2}\left(x\right)\cos2\varphi +0.3153915P_{6}^{6}\left(x\right)\cos6\varphi$$

Although the Popa model [36] can be used to describe the profile broadening, there are other contributions to the peak broadening from the defects as well; for instance, stacking faults, point defects, etc. It is, however, possible to separate the various contributions, if $\langle R_{h}\rangle$ is replaced by an effective radius:

(9) $${\langle R_{h}\rangle }_{eff}^{-1}={\langle R_{h}\rangle }^{-1}+2p_{f}P_{h}$$

where $p_{f}$ is the faulting probability, which must be a refinable parameter, and $P_{h}$ is a determined function of *h*, *k*, *l* according to Warren.

According to the Warren theory [37,38], for an FCC (face-centered cubic) metal, the peak shift and the symmetrical broadening of the peak profiles are proportional to the stacking faults, while the asymmetrical broadening of the peak profiles is related to the twin faults. In Warren theory, it is assumed that the stacking faults of FCC metal with probability α, and the twin faults with probability β, occur independently on the γ_111_ plane. Each close-packed plane γ_111_ is perfect, and the fault densities α and β are both small. Furthermore, the fault densities should be equal for all crystallites. The expression of the peak shift due to the stacking faults follows:

(10) $$\frac{∆Q}{Q}=\frac{45\sqrt{3}\alpha }{\pi^{2}\left(u+b\right)h_{0}^{2}}\sum_{b}\left(\pm \right)L_{0}$$

where Q is the scattering vector, and u, b, h, and $L_{0}$ are the crystal structure-related parameters. For a given crystal structure, the values of $\sum_{b}\left(\pm \right)L_{0}/h_{0}^{2}\left(u+b\right)$ are constants for the corresponding planes. For FCC metal, the values are listed in Table 2.

Since the faulting also acts as a particle size effect that contributes to the peak broadening, the effective crystal dimension D(eff) is considered as [38]:

(11) $$\frac{1}{D\left(\mathrm{eff}\right)}=\frac{1}{D}+\frac{\left(1.5\alpha +\beta \right)}{ah_{0}\left(u+b\right)}\sum_{b}\left|L_{0}\right|$$

From the derivation from peak broadening, only the combined value of faulting probability (1.5α+β), not the individual values of α and β, can be obtained. Nevertheless, the stacking fault probability α can be obtained independently by the peak shift. Furthermore, the twin fault probability β can be determined by the peak asymmetry [38]:

(12) $$\beta =\frac{\sqrt{3}\pi x_{2}\left(y_{1}-y_{2}\right)}{2A}\left\{1+{\left[\frac{1}{D\left(\mathrm{eff}\right)\left(Q_{2}-Q_{0}\right)}\right]}^{2}\right\}$$

Here, two scattering vectors $Q_{1}$ and $Q_{2}$ are chosen in equidistance near the peak center (corresponding to $Q_{0}$), in order to reflect the peak asymmetry. $x_{2}$ is the distance between $Q_{2}$ and $Q_{0}$ on the diffraction profile, $y_{1}$ and $y_{2}$ is the height of the diffraction curve corresponding to the scattering vector $Q_{1}$ and $Q_{2}$, and A is the peak area on the diffraction profile. The detailed description can be found elsewhere [38].

## 3. Results

### 3.1. Microstructure Characterization

**Figure 4:**
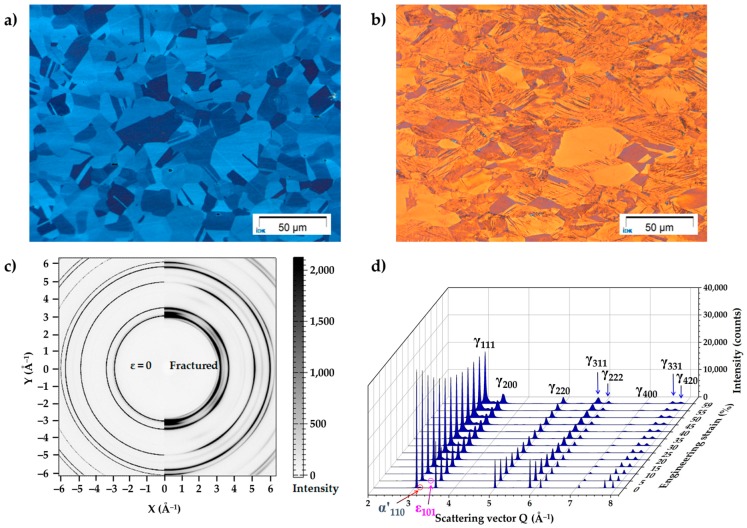
(**a**) Microstructure of the Fe-17Mn-1.5Al-0.3C steel before tensile test [4]; (**b**) microstructure of the steel after tensile test [4]; (**c**) two-dimensional diffraction pattern from the steel before and after the tensile test; and (**d**) the SYXRD profiles of the integrated intensities at different engineering strain levels.

Figure 4a shows the microstructure of the tensile test sample prior to the deformation. The undeformed microstructure is full austenite with some annealing twins and the average grain size is about 17 µm [4]. After deformation at room temperature, parallel striations and fine inordinate substructures coexist in the microstructure, as shown in Figure 4b, which indicates a mixed deformation mode [4]. Figure 4c shows the two-dimensional diffraction patterns before and after the tensile test, and the different Debye-Scherrer rings donate the diffraction on the various lattice planes of γ-austenite, α’-martensite, and ε-martensite phases. The diffraction pattern of the steel in the initial state (before deformation, ε = 0) displays isotropic Debye-Scherrer rings, which indicates a randomly-textured polycrystalline material. In contrast, the diffraction pattern reveals anisotropic Debye-Scherrer rings, donating a deformation texture. The current study focuses on the dynamic microstructure evolution during a tensile test, and the harmonic texture model [35] was used in the refinement, in which the sample symmetry was assumed as fiber. For the detailed texture analysis one can perform the texture measurements by the systematic rotation of the sample to cover all possible lattice planes into refection condition. However, the interruption of tensile test results in the stress relaxation effect, which might lead to the inaccuracy of the microstructure analysis.

The SYXRD profiles of the integrated intensity over scattering vector *Q* at different strain levels are plotted in Figure 4d. It can be seen that there is a small amount of α’-martensite in the initial state, which might result from the sample preparation process by grinding. Thanks to the high energy of the synchrotron X-ray, the early stage of ε-martensite transformation can be monitored, as shown in Figure 4d. In addition, the diffraction peaks show strongly broadening behavior, which is due to the nucleation and accumulation of defects, e.g., dislocations [39]. The previous study by Yan et al. [39] concluded that broadening is ascribed to slip as a deformation mechanism. Apart from the slip mechanism, the TRIP and TWIP effects are focused in the present study. In order to quantitatively determine and analyze the microstructure evolution during the tensile deformation, the diffraction profiles from the initial state (0 engineering strain) up to 57.5% engineering strain, with a 2.5% engineering strain step and the SYXRD profile at fracture (59% engineering strain), were analyzed by the Rietveld refinement method.

**Figure 5:**
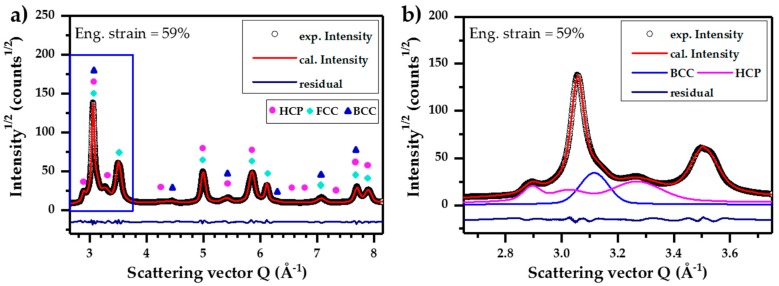
The SYXRD profile of the Fe-17Mn-1.5Al-0.3C steel at fracture (ε = 59%) fitted by the Rietveld refinement method using MAUD software: (**a**) full profile and (**b**) zoom-in of the region marked in (a) showing the fitted curves of the individual phases.

As an example, the fitted curve of diffraction profile at the engineering strain 59% (fractured state) is displayed in Figure 5. In Figure 5a, the circles indicate the measured SYXRD intensity (square root) as a function of the scattering vector *Q*. The red line shows the fitted curve by Rietveld refinement method and the dark blue line is the residual, namely, the intensity difference between the experimental measurement and the Rietveld refinement. It can be seen that residual is small and the calculated curve by the Rietveld refinement methods fits the experiment date commendably. According to crystallography, the diffraction peaks can be indexed as FCC, BCC (body-centered cubic), and HCP (hexagonal close packed) crystal structures, which are correlated with the γ-austenite phase, α‘-martensite phase, and ε-martensite phase, respectively. Figure 5b shows the diffraction profile from 2.65 Å^−1^ to 3.75 Å^−1^ in scattering vector *Q*. The light blue curve and pink curve correlate with the diffraction peaks of BCC structure (α’-martensite) and HCP structure (ε-martensite), correspondingly. The diffraction peaks are indexed correspondingly to the crystallographic planes ε_100_, γ_111_, ε_002_, α’_110_, ε_110_, and γ_200_ with the increase of Bragg’s angle. The contribution of different phases to the diffraction profile can be easily distinguished by the Rietveld refinement method. The microstructure information in the materials, such as phase fraction, lattice parameter, microstrain, crystallite size, stacking fault probability, and twinning fault probability, can be obtained by the Rietveld refinement. The microstructure evolution with the increase in deformation strain will be discussed below.

#### 3.1.1. Phase Evolution

**Figure 6:**
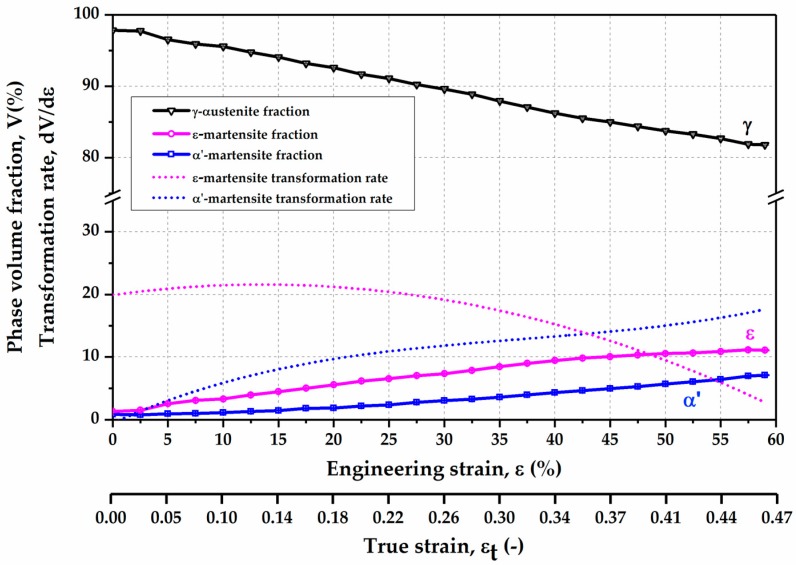
Phase volume fraction and corresponding phase transformation rate as a function of tensile strain in the Fe-17Mn-1.5Al-0.3C steel measured by SYXRD.

The phase volume fractions of γ-austenite, ε-martensite, and α’-martensite are plotted as a function of the engineering strain, as illustrated in Figure 6. The volume fraction of γ-austenite phase progressively declines with the increase in the deformation degree, while the volume fractions of ε-martensite and α’-martensite increase with the increase in the deformation strain. This indicates the occurrence of strain-induced phase transformation in the present material upon deformation. The ε-martensite volume fraction continuously rises up to 11.1 vol % with the increase in deformation degree upon fracture. However, in the low engineering strain range of 0 to 10%, the α’-martensite volume fraction manifests only a slight change, and then it increases successively up to 7.1 vol % up to the fracture of the sample.

In order to further analyze the change of the volume fractions of different phases during the deformation, the phase transformation rates of ε-martensite and α’-martensite are estimated by calculating the first derivative of the phase volume fractions over engineering strain. The estimated transformation rates of ε-martensite and α’-martensite are shown in Figure 6 as dot lines. The transformation rate of ε-martensite shows a much higher value (~20%) in the beginning of the deformation than that of α’-martensite, and it increases continuously with the increase in engineering strain up to approximately 15%. Then it drops with the increase in engineering strain. In contrast, α’-martensite exhibits a lower transformation rate (<2%) in the beginning of deformation. Then, the transformation rate of α’-martensite increases progressively up to the fracture of the sample. At about 42.5% engineering strain (corresponding to true strain 0.35), the transformation rate of α’-martensite is equal to that of ε-martensite. Below 42.5% engineering strain, the transformation rate of ε-martensite is higher than that of α’-martensite, the strain-induced ε-martensite transformation is the dominant deformation mechanism. On the contrary, the transformation rate of α’-martensite is superior to that of ε-martensite when the engineering strain is above 42.5%. The strain-induced α’-martensite transformation predominates at the higher strain level.

#### 3.1.2. Planar Defects Evolution

**Figure 7:**
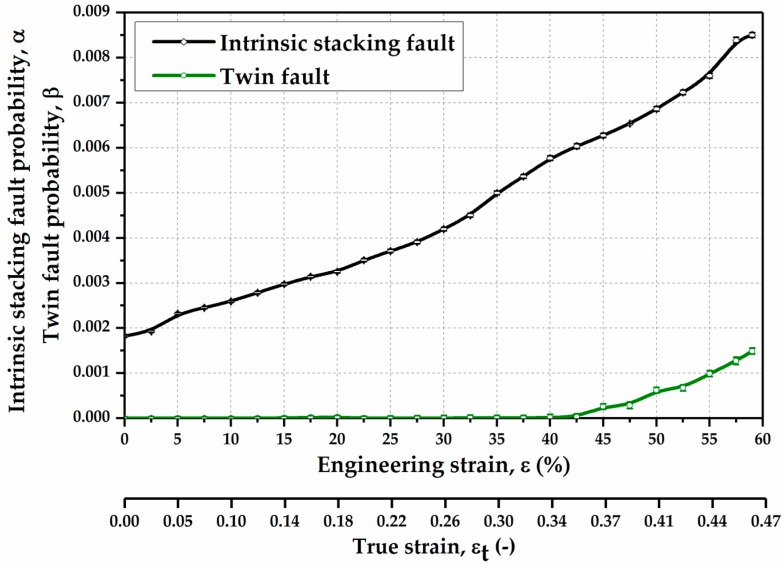
Intrinsic stacking fault probability and twin fault probability as a function of strain in the Fe-17Mn-1.5Al-0.3C steel measured by SYXRD.

The evolution of planar defects such as intrinsic stacking fault and twin fault with the increase in engineering strain in the Fe-17Mn-1.5Al-0.3C steel is illustrated in Figure 7. The intrinsic stacking fault probability shows a successive increase with the progress of deformation. Prior to deformation, the intrinsic stacking fault probability is about 0.0018. During the deformation, lots of stacking faults are generated, in the way of the ordered stacking sequence changing into a wrong stacking sequence. Therefore, the stacking fault probability increases up to 0.0085 upon the fracture of the sample. The twin fault probability remains a relatively low value (below 0.00001) at the low strain level (engineering strain <42.5%), and it rises strongly from the engineering strain 42.5% (corresponding to the true strain 0.35). The twin fault probability is approximately 0.0015, when the material is fractured. It is indicated that the TRIP effect is not the only deformation mechanism when the engineering strain is higher than 42.5%. In addition, the TWIP effect is also activated at the higher deformation degree.

### 3.2. SFE Evaluation

Compared with other mechanical and physical measurements, the SYXRD method is statistically more meaningful for the SFE measurement due to its large detection volume on the order of mm^3^. In the determination of SFE by XRD method, the Reed and Schramm’s equation [40,41] has been widely used, which indicates that the SFE is proportional to the mean square strain and inversely proportional to the stacking fault probability:

(13) $$\gamma =\frac{K_{111}\omega_{0}G_{\left(111\right)}a_{0}A^{-0.37}}{\pi \sqrt{3}}\frac{{\langle \varepsilon_{50}^{2}\rangle }_{111}}{\alpha }$$

where, $K_{111}\omega_{0}$ is proportionality constant and it is 6.6, $G_{\left(111\right)}$ is the shear modulus in the γ_111_ fault plane and it is 65 GPa for austenitic steels [41], $a_{0}$ is the lattice parameter, A is the Zener anisotropy parameter and it is 3.43 [41], ${\langle \varepsilon_{50}^{2}\rangle }_{111}$ is the microstrain averaged over a column 50 Å in the γ_111_ fault plane, and α is the stacking fault probability. Therefore, the SFE of the material can be indirectly measured using XRD by calculating the ratio of the mean square strain and stacking fault probability.

**Figure 8:**
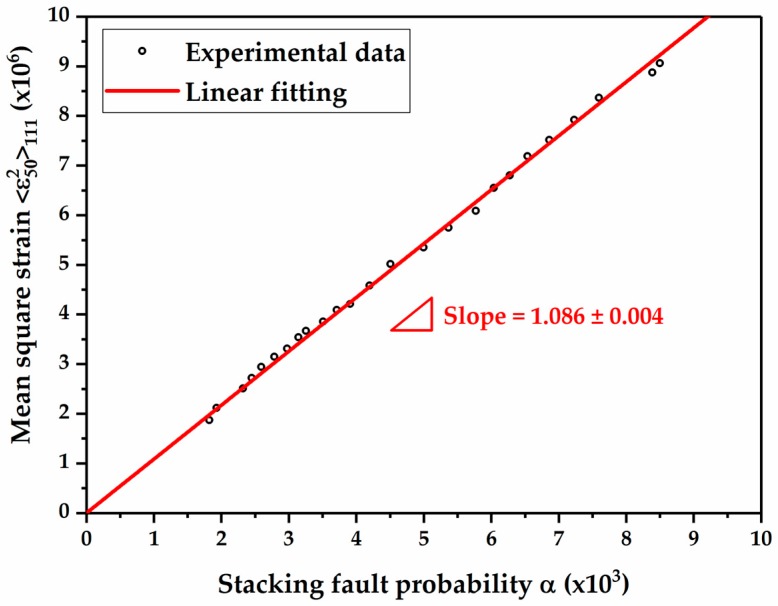
The plot of mean square strain vs. stacking fault probability in the Fe-17Mn-1.5Al-0.3C steel corresponding to various tensile strain levels.

The mean square strain versus the stacking fault probability measured at each deformation stage is plotted as circles in Figure 8. Both the mean square strain and stacking fault probability show the positive correlation with the deformation degree. Since the SFE is an intrinsic parameter of a material, and it is a function of material’s chemistry and temperature, the ratio of the mean square strain to stacking fault probability should be a constant value despite the accumulated strain [42]. Hence, the SFE of the Fe-17Mn-1.5Al-0.3C steel can be determined by the slope of the linear regression in Figure 8. The slope of the fitted line is 1.086 with an error bar of 0.004. Therefore, the SFE of the material is evaluated to be 19.3 mJ/m^2^, with a minor deviation of 0.1 mJ/m^2^.

In addition, the SFE of high-Mn steels can be calculated by a thermodynamic model as well. According to thermodynamics, the SFE of high-Mn steels is associated with the Gibbs free energy difference between two atomic layers of HCP and FCC structures [43]. The SFE can be formulated as follows:

(14) $$\gamma_{SFE}=2\rho ∆G_{eff}^{\gamma \to \varepsilon }+2\sigma^{\gamma /\varepsilon }$$

(15) $$\rho =\frac{4}{\sqrt{3}}\frac{1}{a^{2}N}$$

where, $\gamma_{SFE}$ is the stacking fault energy; ρ is the molar surface density along the {111} planes; $G_{eff}^{\gamma \to \varepsilon }$ is the Gibbs free energy due to the transformation of γ-austenite to ε-martensite, which consists of two parts, the chemical contribution and the magnetic contribution; $\sigma^{\gamma /\varepsilon }$ is the surface energy of the γ-austenite and ε-martensite interface; a is the lattice parameter and N is the Avogadro constant.

In the thermodynamic model, the SFE is described as a function of temperature and material’s chemistry. The calculation was based on a subregular solution thermodynamic model proposed by Saeed-Akbari et al. [9]. The SFE was calculated by using the actual Fe, Mn, Al, and C contents listed in Table 1. The value of γ-austenite/ε-martensite interfacial energy 10 mJ/m^2^ was used. The SFE of the Fe-17Mn-1.5Al-0.3C steel is about 18.9 mJ/m^2^ at temperature 300 K.

### 3.3. Tensile Properties

**Figure 9:**
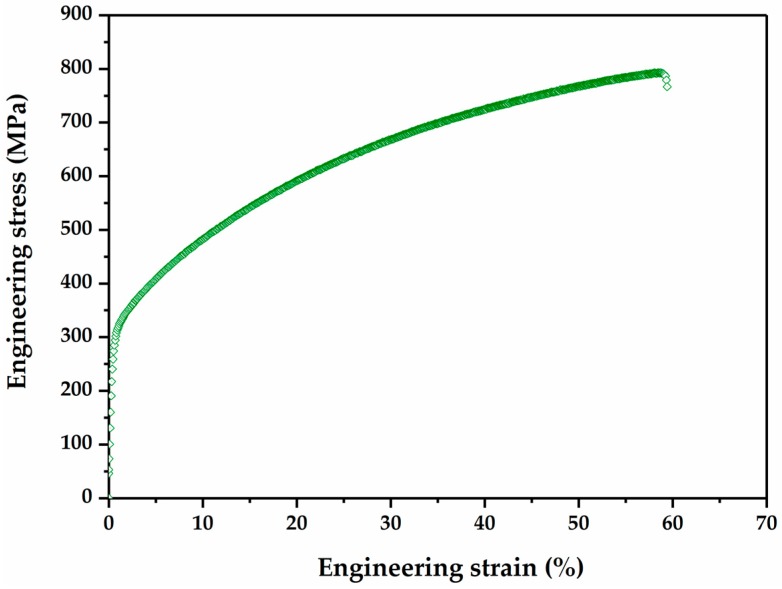
Engineering stress–engineering strain curve of the Fe-17Mn-1.5Al-0.3C steel tested at room temperature and a strain rate of 1 × 10^−3^ s^−1^.

Figure 9 displays engineering strain–engineering stress curve of the Fe-17Mn-1.5Al-0.3C steel. The present high-Mn steel possesses a good combination of high strength and superior ductility. The mechanical properties of the Fe-17Mn-1.5Al-0.3C steel are listed in Table 3. The engineering strain—engineering stress curve shows continuous yielding, and the proof stress $\sigma_{0.2}$ is 288 MPa. The ultimate tensile strength is 743 MPa at the uniform elongation 58%. The Eco-Index (the product of uniform elongation and ultimate tensile strength) gains 43 GPa % for this steel, which indicates the high energy absorption potential of the Fe-17Mn-1.3Al-0.3C steel. There exists no pronounced serration phenomenon on the engineering strain–engineering stress curve, which is a typical feature for Fe-Mn-C high-Mn steels, and this might be due to the addition of Al in the investigated steel [44].

**Figure 10:**
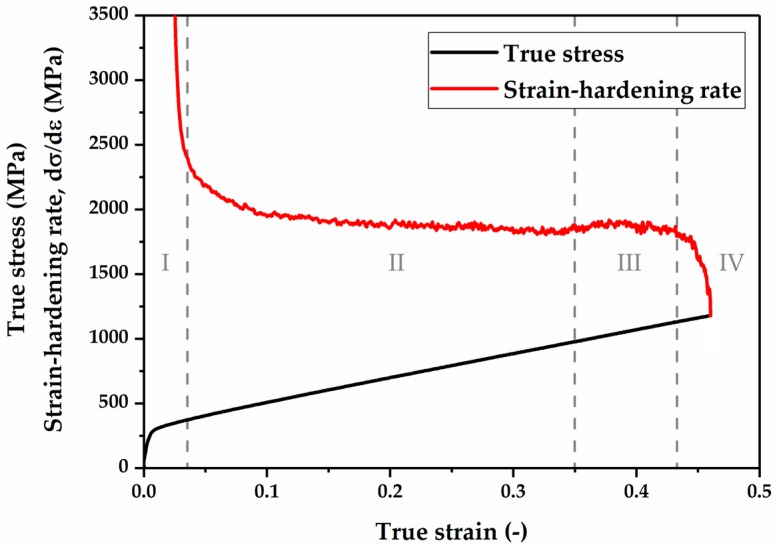
True strain–true stress curve and strain-hardening rate of the Fe-17Mn-1.5Al-0.3C steel tested at room temperature and a strain rate of 1 × 10^−3^ s^−1^.

The true strain–true stress curve and strain-hardening rate of the Fe-17Mn-1.5Al-0.3C high-Mn steel are shown in Figure 10, and the plastic deformation region could be divided into four different stages (stage I–stage IV). At the beginning of plastic deformation (stage I), the steel manifests an intensely high strain-hardening rate and it is followed by a dramatic decrease in the strain-hardening rate up to the true strain 0.025. After the pronounced decline, the strain-hardening rate decreases slightly with the increase in the true strain and reaches a constant value up to the true strain 0.35, which is defined as stage II. In stage III, the strain-hardening rate increases slightly in the true strain range of 0.35–0.43. In the final stage (stage IV), the strain-hardening rate rapidly decreases and the specimen fractures.

## 4. Discussion

The investigated Fe-17Mn-1.5Al-0.3C steel reveals an extraordinary combination of high ultimate tensile strength of 743 MPa and superior ductility of approximately 60%, which correlates with the extremely high strain-hardening rate about 2000 MPa during deformation. Based on the results obtained in the present study, the microstructure evolution and strengthening mechanisms in the Fe-17Mn-1.5Al-0.3C steel during plastic deformation are discussed below.

**Figure 11:**
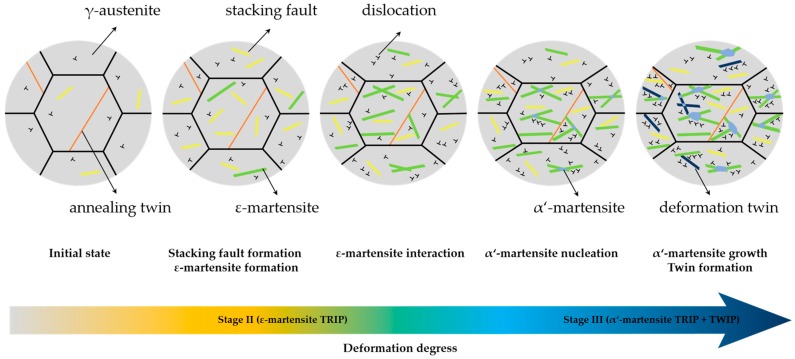
Schematic sketches of the microstructure evolution in Fe-17Mn-1.5Al-0.3C TRIP/TWIP steel during plastic deformation.

The SFE is believed to be a crucial parameter determining the microstructure evolution and deformation mechanisms in high-Mn austenitic steels [4,9,11]. The evaluation of the SFE of the Fe-17Mn-1.5Al-0.3C steel by the SYXRD measurements, as well as by the thermodynamic calculation, resonate well. It is confirmed that the SFE of the Fe-17Mn-1.5Al-0.3C steel is about 19 mJ/m^2^. Previous studies pointed out that the TRIP-TWIP transition occurs at the SFE value of ~20 mJ/m^2^ [4,11]. The coexistence of the strain-induced martensite and the deformation twins detected by SYXRD during tensile deformation confirms the mixture of TRIP and TWIP mechanisms during tensile deformation in the studied material. Besides, the The combination of the different microstructural features, including deformation-induced phases (Figure 6), stacking faults, and twins (Figure 7), enables us to disentangle the underlying deformation mechanisms in the steel (Figure 11).

In stage I (Figure 10), the steel manifests an intensely high strain-hardening rate and it is followed by a dramatic decrease in the strain-hardening rate. Gurierrez-Urrutia et al. [12] reported a similar strain-hardening rate behavior at the beginning of the deformation in Fe-22Mn-0.6C TWIP steel. A similar feature was also found in a low SFE FCC material [45]. The high strain-hardening rate at the beginning of the plastic deformation results from the pile-up of dislocations at the grain boundaries. The drop of the strain-hardening rate at this stage might be attributed to the occurrence of dynamic recovery processes, accompanied by the rearrangement and annihilation of dislocations of the opposite signs.

In stage II, the SYXRD profile analysis reveals that the deformation-induced γ-austenite to ε-martensite transformation with a high transformation rate is responsible for the remaining high strain-hardening rate. The SYXRD investigation shows that a large number of stacking faults are generated during deformation. Stacking faults in the γ-austenite are formed by the movement of Shockley partial dislocations with Burgers vector of 1/6 <112> [17,46]. When Shockley partial dislocations are formed and extend on every second {111} crystallographic plane in γ-austenite, the stacking faults overlap to form ε-martensite [47,48]. It is believed that the ε-martensite can effectively contribute to the increase in the strain-hardening rate in high-Mn steels [17,49,50], because the deformation-induced phase transformation generates new boundaries as additional obstacles impeding the dislocation movement [51]. In addition, the intersections of ε-martensite plates can play a crucial role in the nucleation of α’-martensite [18,52,53]. However, in stage II, ε-martensite transformation exhibits a transformation rate superior to that of α’-martensite. The ε-martensite TRIP effect, combined with dislocation slipping, is the dominant deformation mechanism in stage II (Figure 6).

In contrast, in stage III, the quantitative phase analysis indicates that α’-martensite manifests a higher transformation rate than that of ε-martensite (Figure 6). The formation and growth of α’-martensite are more dominant in this stage. The continuous increase in the ε-martensite population results in a considerable number of ε-martensite intersections, which can promote α’-martensite nucleation effectively. In addition, during further deformation, α’-martensite grows into large plates by coalescence of α’-martensite embryos [52]. Consequently, a large amount of α’-martensite is induced by deformation. Furthermore, the twin fault probability increases strongly in this stage. From a crystallographic point of view, the formation of a deformation twin can be explained by the stacking faults taking place on every close-packed {111} plane in γ-austenite [47,48]. The presence of deformation twins at this stage might be attributed to the increase in the SFE locally. The heat generated during plastic deformation results in a rise in the local temperature at high deformation degree, which might lead to an increase in the SFE locally above 20 mJ/m^2^ (the TRIP-TWIP transition borderline). Nevertheless, the SYXRD probes a large detection volume of the material; the average SFE value (19.3 mJ/m^2^) might not be strongly affected by the increase in the local temperature, as shown in Figure 8. In addition, the intersection of deformation twins can also act as nucleation sites of α’-martensite [52,53]. Therefore, in stage III, both the intersections of ε-martensite plates and of deformation twins facilitate the α’-martensite nucleation. The elevated deformation twins and phase boundaries resulting from the strain-induced twinning and phase transformation act as obstacles against the dislocation slip, thus leading to an improved strain-hardening rate. A combination of the α’-martensite predominant TRIP effect and the TWIP effect in stage III effectively increases the strain-hardening rate. In stage IV, the further strain-hardening potential was exhausted, which led to a fracture of the specimen.

## 5. Conclusions

An in situ tensile test tracked by high-energy synchrotron X-ray diffraction was carried out to investigate the strain-hardening behavior of a high-Mn steel. The Fe-17Mn-1.5Al-0.3C high-Mn steel exhibits a high ultimate tensile strength of 743 MPa with a superior uniform elongation of 58%, and a high strain-hardening rate of approximately 2000 MPa. During plastic deformation, this steel manifests a mixture of TRIP and TWIP mechanisms. The remaining high strain-hardening rate in stage II (0.025–0.35) results from the ε-martensite dominant TRIP effect; the increase in the strain-hardening rate in stage III (0.35–0.43) is attributed to the synergy of α’-martensite dominant TRIP effect and TWIP effect. An evaluation of the SYXRD results suggests that Fe-17Mn-1.5Al-0.3C steel has a stacking fault energy around 19.3 mJ/m^2^, which indicates the synergic effect of TRIP and TWIP, as well.

## Figures and Tables

**Table 1:** Chemical composition of the Fe-17Mn-1.5Al-0.3C steel.

Element	C	Si	Mn	P	S	Al	Fe
wt %	0.32	0.03	17.24	0.009	0.008	1.75	Bal.

**Table 2:** Constants for powder pattern of faulted FCC metals [38].

hkl	111	200	220	311	222	400
$\frac{\sum_{b}\left(\pm \right)L_{0}}{h_{0}^{2}\left(u+b\right)}$	1/4	−1/2	1/4	−1/11	−1/8	1/4
$\frac{\sum_{b}\left|L_{0}\right|}{h_{0}^{2}\left(u+b\right)}$	$\sqrt{3}/4$	1	$1/\sqrt{2}$	$\frac{3}{2}\sqrt{11}$	$\sqrt{3}/4$	1

**Table 3:** Mechanical properties of the Fe-17Mn-1.5Al-0.3C steel.

Steel	$\sigma_{0.2}$ (MPa)	$\sigma_{u}$ (MPa)	$\varepsilon_{u}$ (%)	$\varepsilon_{f}$ (%)	Eco-Index (GPa %)
Fe-17Mn-1.5Al-0.3C	288	743	58	59	43
